# A phase 2, open-label, multicenter study of ixazomib plus lenalidomide and dexamethasone in adult Japanese patients with relapsed and/or refractory multiple myeloma

**DOI:** 10.1007/s10147-021-02030-7

**Published:** 2021-10-02

**Authors:** Shinsuke Iida, Tohru Izumi, Takuya Komeno, Yasuhito Terui, Takaaki Chou, Takashi Ikeda, Deborah Berg, Shinichi Fukunaga, Kenkichi Sugiura, Makoto Sasaki

**Affiliations:** 1 Department of Hematology and Oncology, Nagoya City University Graduate School of Medical Sciences, Kawasumi 1, Mizuho-cho, Mizuoho-ku, Nagoya-shi, Aichi Japan; 2 Department of Hematology, Tochigi Cancer Center, Utsunomiya, Tochigi Japan; 3 Department of Hematology, Sendai Medical Center, Sendai, Miyagi Japan; 4 Department of Hematology, National Hospital Organization Mito Medical Center, Ibaraki, Japan; 5 Department of Hematology Oncology, Cancer Institute Hospital of Japanese Foundation for Cancer Research, Koto, Tokyo Japan; 6 Department of Hematology, Saitama Medical University Hospital, Moroyama-machi, Saitama, Japan; 7 Department of Internal Medicine, Niigata Cancer Center Hospital, Niigata, Japan; 8 Niigata Kenshin Plaza, General Incorporated Foundation, Health Medicine Prevention Association, Niigata, Japan; 9 Division of Hematology and Stem Cell Transplantation, Shizuoka Cancer Center, Nagaizumi, Shizuoka Japan; 10 Clinical Research and Development, Takeda Pharmaceutical Company Limited, Osaka, Japan; 11 Oncology Therapeutic Area Unit for Japan and Asia, Takeda Pharmaceutical Company Limited, Osaka, Japan; 12 Statistical & Quantitative Sciences, Data Sciences Institute, Research & Development, Takeda Pharmaceutical Company Limited, Osaka, Japan; 13 Division of Hematology, Department of Internal Medicine, Juntendo University School of Medicine, Tokyo, Japan

**Keywords:** Ixazomib, RRMM, Efficacy, Safety, Japanese

## Abstract

**Background:**

TOURMALINE-MM1 was a global study that demonstrated a significant improvement in progression-free survival with ixazomib plus lenalidomide and dexamethasone compared with placebo plus lenalidomide and dexamethasone, in patients with relapsed and/or refractory multiple myeloma. The current study was conducted to evaluate further the efficacy and safety of ixazomib plus lenalidomide and dexamethasone in Japanese patients.

**Methods:**

This phase 2, open-label, single-arm, multicenter study enrolled patients aged ≥ 20 years with relapsed and/or refractory multiple myeloma at 16 sites in Japan. Patients refractory to lenalidomide or proteasome inhibitor-based therapy at any line were excluded. The primary endpoint was the rate of very good partial response or better in the response-evaluable analysis set. Secondary endpoints were progression-free survival, overall response rate, duration of response, time to progression, overall survival and safety.

**Results:**

In total, 34 patients were enrolled. The rate of very good partial response or better was 50.0% (95% confidence interval 31.9–68.1) and the overall response rate was 84.4% (95% confidence interval 67.2–94.7). Median progression-free survival was 22.0 months (95% confidence interval 17.3–not evaluable) and median overall survival was not estimable. The safety profile of ixazomib plus lenalidomide and dexamethasone in this study was similar to that in the TOURMALINE-MM1 study.

**Conclusions:**

The efficacy and safety of ixazomib plus lenalidomide and dexamethasone in Japanese patients with relapsed and/or refractory multiple myeloma are comparable with reported TOURMALINE-MM1 study results.

**Clinicaltrials.gov identifier:**

NCT02917941; date of registration September 28, 2016.

**Supplementary Information:**

The online version contains supplementary material available at 10.1007/s10147-021-02030-7.

## Introduction

Multiple myeloma (MM) is a malignant disease in which monoclonal plasma cells proliferate, mainly in the bone marrow. MM causes increases in monoclonal immunoglobulin (M-protein) production by myeloma cells, hematopoietic deterioration, bone destruction, hypercalcemia and renal failure. MM constitutes approximately 1% of all reported neoplasms and approximately 13% of hematologic cancers worldwide [1]. In Japan, the National Cancer Center estimated there would be 7,800 new cases of MM, with approximately 4500 deaths in 2019 [2].

The treatment landscape has shifted from autologous stem cell transplantation (ASCT) being the mainstay of therapy, to its combination with novel agent-based induction regimens and post-ASCT consolidation and maintenance treatments [3]. While historic treatment approaches focused on the use of cytotoxic drugs such as alkylating agents, anthracyclines and corticosteroids, the introduction of the first-in-class proteasome inhibitor (PI), bortezomib and immunomodulatory drugs (IMiDs) such as thalidomide and lenalidomide have improved treatment outcomes [4–7]. PIs and IMiDs currently remain the backbone of therapy throughout the MM treatment pathway [8, 9]. Monoclonal antibody drugs also play an important role [10].

Although some data have suggested an increasing cure fraction rate in front-line patients, MM is still generally regarded as incurable [11]. Most patients receive multiple lines of therapy, including combination regimens, over the course of their disease [12].

In an effort to further target the increased proteasome activity known to occur in MM and other cancers, ixazomib, a small molecule 20S PI, was developed. In contrast to bortezomib, ixazomib has demonstrated a faster dissociation rate from the proteasome; improved pharmacokinetics and pharmacodynamics, which may result in enhanced tumor penetration; and antitumor activity in a broader range of tumor xenografts [13]. The clinical benefit of ixazomib has been studied previously and ixazomib is being developed globally as a treatment option for relapse/refractory (RR)MM, newly diagnosed (ND)MM, maintenance monotherapy for MM and relapsed or refractory systemic light-chain amyloidosis [14–17]. In Japan, ixazomib is approved for the treatment of RRMM and more recently was approved as maintenance monotherapy.

The pivotal TOURMALINE-MM1 (MM1) study was a phase 3 global, randomized, double-blind, placebo-controlled study that evaluated the efficacy and safety of ixazomib combined with lenalidomide and dexamethasone (LenDex) versus placebo combined with LenDex, in patients with RRMM who had received at least one prior therapy [17]. The efficacy results of the primary analysis for the overall population demonstrated a statistically significant and clinically meaningful prolongation in the primary endpoint of progression-free survival (PFS) with the ixazomib plus LenDex regimen compared with the placebo plus LenDex regimen (median PFS of 20.6 months versus 14.7 months, respectively [hazard ratio 0.742; *p* = 0.012]), as assessed by an independent review committee [17].

In addition, the ixazomib plus LenDex regimen provided clinical benefit, as demonstrated by significant improvements in complete response (CR) rate, overall response rate (ORR) and rate of very good partial response (VGPR) or better (VGPR + CR), and longer disease control, as demonstrated by a significant improvement in time to progression (TTP) and a longer duration of response (DOR). Ixazomib in combination with LenDex has thus been shown to be an efficacious regimen [17].

However, the PFS data for the Japanese subpopulation in the MM1 study was limited [17]; hence, consistency with the overall population could not be concluded, and efficacy in Japanese patients remained to be confirmed.

The current study evaluated the efficacy and safety of ixazomib when administered with LenDex in Japanese patients with RRMM. In relapsed or refractory settings, significantly longer PFS or TTP have been demonstrated in patients with VGPR or better, compared with those with partial response (PR) [18]. Therefore, the endpoint of VGPR + CR rate is often clinically correlated with PFS. Hence, the primary objective of this study was to determine the rate of VGPR or better in the response-evaluable analysis set, which was agreed upon in a consultation meeting between the study sponsor and the Pharmaceuticals and Medical Devices Agency of Japan.

## Materials and methods

### Study design

This was a phase 2, open-label, single-arm, multicenter study, with patients enrolled at 16 study sites in Japan. Response was assessed according to the International Myeloma Working Group criteria every 4 weeks until progressive disease (PD) [19]. All patients were followed up for survival after progression, and patients were contacted every 12 weeks until death or termination of the study. Patients attended an end of treatment visit approximately 30 days after receiving their last dose of any study drug (ixazomib, lenalidomide or dexamethasone) and continued to be followed up for other assessments. Patients discontinuing study treatment prior to PD continued to be assessed for PD during the PFS follow-up portion of the study. Primary analysis was conducted approximately 12 months after the last enrollment, and the final analysis was conducted after the final database lock, approximately 24 months after the last enrollment.

This study was conducted in compliance with Good Clinical Practice (GCP), Good Post-marketing Study Practice (GPSP) and all applicable local regulations and guidelines. All study-related documents were reviewed and approved by the local or central institutional review boards of all study sites. This study was also conducted in accordance with the Declaration of Helsinki, and the International Council for Harmonisation of Technical Requirements for Pharmaceuticals for Human Use (ICH) Harmonised Tripartite Guideline for GCP and GPSP, and all applicable regulations. Objectives and potential risks and benefits were explained to patients using the informed consent form approved by the institutional review board, with each patient having signed and dated the form before screening.

### Patient selection

Important inclusion criteria were (1) Japanese patients ≥ 20 years with diagnosed MM and measurable disease with serum M-protein ≥ 1 g/dl, urine M-protein ≥ 200 mg/24 h or abnormal serum free light chain (FLC) ratio with involved FLC level ≥ 10 mg/dl and who were relapsed and/or refractory after receiving one to three prior therapies; (2) patients who had an Eastern Cooperative Oncology Group (ECOG) performance status of 0, 1 or 2. Patients who had received autologous transplants were also eligible for inclusion. Additional inclusion criteria are described in Online Resource 1.

An important exclusion criterion was patients who were refractory to lenalidomide or PI-based therapy at any line. Additional exclusion criteria are described in Online Resource 1.

### Treatment

Patients received 4 mg of ixazomib on days 1, 8 and 15 plus lenalidomide (25 mg) on days 1 through 21, and dexamethasone (40 mg) on days 1, 8, 15 and 22 of a 28-day cycle. Patients with a low creatinine clearance of < 60 ml/min received a reduced lenalidomide dose of 10 mg once daily. Patients continued to receive treatment until progressive disease or unacceptable toxicity, whichever came first.

### Endpoints

The primary endpoint was the rate of VGPR + CR in the response-evaluable analysis set. The secondary endpoints were PFS, ORR, DOR, TTP, safety and overall survival (OS). The definitions of secondary endpoints are described in Online Resource 2.

### Statistical analysis

For the VGPR + CR rate and ORR, two-sided 95% confidence intervals (CIs) were calculated in the response-evaluable analysis set and the full analysis set (FAS). The response-evaluable population was defined as patients who received at least one dose of ixazomib, had measurable disease at baseline and had at least one post-baseline response assessment. For PFS, OS and TTP, Kaplan–Meier estimates (and the 25th, 50th [median] and 75th percentiles, if estimable) were calculated with their two-sided 95% CIs in the FAS. For DOR, the Kaplan–Meier estimates was calculated as well for responders. Based on the results of the MM1 study, a sample size of 27 was required to provide a point estimate of 48.1% for the expected VGPR + CR rate, which was higher than the threshold rate of 39.0% with 80% probability. Assuming a dropout rate of 10%, the target number of patients for this study was set to 30. All analyses were conducted with SAS version 9.2.

## Results

### Patient background

Patient demographics and baseline characteristics are described in Table 1. Of the 34 patients in the FAS, the median age was 67 years. Median time from initial diagnosis was 44.4 months, with 71% of MM diagnosed being of the IgG type.

**Table 1:** Patient demographics and baseline characteristics in the full analysis set

	Ixazomib + LenDex *N* = 34
Age (years)	
Median	67
Min, max	40, 78
Sex (*n*, %)	
Male	19 (56)
Female	15 (44)
Baseline body surface area (m^2^)	
Median	1.62
Min, max	1.20, 2.03
Time since initial diagnosis to first dose at study entry (months)	
Median	44.4
Min, max	10, 176
Type of myeloma at initial diagnosis (*n*, %)	
IgG	24 (71)
IgA	4 (12)
Bence-Jones	5 (15)
Light chain only	1 (3)
ISS stage for myeloma at study entry (*n*, %)	
I	28 (82)
II	5 (15)
III	1 (3)
Creatinine clearance (ml/min) (*n*, %)	
30 to < 60	10 (29)
60 to < 90	15 (44)
90 to max	9 (26)
Baseline ECOG performance status (*n*, %)	
0	25 (74)
1	9 (26)
Prior therapy (*n*, %)	
Lines of prior therapy	
1	21 (62)
2	12 (35)
3	1 (3)
Patient population categories	
Relapsed patients^a^	31 (91)
Refractory patients^b^	3 (9)
Patients with ASCT	23 (68)
Prior IMiD therapy	
Exposed	12 (35)
Thalidomide^c^	5 (15)
Lenalidomide^c^	8 (24)
Naive	22 (65)
Prior PI therapy	
Exposed	31 (91)
Bortezomib^d^	30 (88)
Carfilzomib^d^	2 (6)
Naive	3 (9)
Cytogenetics	
Cytogenetics results	
High risk^e^	11 (32)
Standard	21 (62)
Not available	2 (6)
Chromosomal abnormalities	
Del 13 or 13q	15 (44)
Del 17 or 17p	4 (12)
t(4;14)	7 (21)
t(11;14)	2 (6)
t(14;16)	1 (3)

*ASCT* autologous stem cell transplantation, *ECOG* Eastern Cooperative Oncology Group, *IMiD* immunomodulatory drug, *PI* proteasome inhibitor, *ISS* International Staging System, *LenDex* lenalidomide and dexamethasone

^a^Relapsed was defined as patients who relapsed from at least one previous treatment but were not refractory to any previous treatment

^b^Refractory was defined as patients who were refractory to at least one previous treatment but did not relapse from any previous treatment

^c^One patient had been exposed to both thalidomide and lenalidomide

^d^One patient had been exposed to both bortezomib and carfilzomib

^e^High-risk cytogenetic abnormalities that were defined as containing t(4;14), t(14;16) or del(17p); cutoff values for defining the presence of t(4;14), t(14;16) and del(17p) were 3%, 3% and 5% positive cells, respectively

At study entry, 82% of patients were International Staging System stage I, and 74% had an ECOG performance status of 0. Additionally, 29% of patients had a creatinine clearance of 30 to < 60 ml/min, 62% of patients only had one prior line of therapy, 91% of patients had relapsed disease and 9% of patients had refractory disease.

Overall, 68% of patients had undergone ASCT with a median time of 38.8 months since the time of last transplantation to the first dose at study entry. 91% of patients had previous exposure to PIs and 35% to IMiDs. 32% of patients had high-risk cytogenetic abnormalities, whereby cut-offs were 5% positive cells for del(17p), 3% for t(4;14) and 3% for t(14;16), of cells testing positive for these abnormalities.

### Exposure

The study drug exposure is described in Table 2. The median number of treatment cycles for all study drugs was 20, with a median relative dose intensity of 86.0% for ixazomib, 81.6% for lenalidomide and 91.7% for dexamethasone.

**Table 2:** Study drug exposure in the safety analysis set

	Ixazomib + LenDex *N* = 34			
	Ixazomib	Lenalidomide	Dexamethasone	Combination
Number of treatment cycles^a^				
Mean (standard deviation)	17.8 (9.3)	17.8 (9.3)	17.8 (9.3)	17.8 (9.3)
Median	20.0	20.0	20.0	20.0
Min, max	1, 32	1, 32	1, 32	1, 32
Relative dose intensity (%)				
Mean (standard deviation)	85.4 (12.8)	78.2 (18.7)	76.5 (25.0)	**–**
Median	86.0	81.6	91.7	**–**
Min, max	59, 100	40, 100	23, 100	**–**

*LenDex* lenalidomide and dexamethasone

^a^A treatment cycle was defined as a cycle in which the patient received any amount of ixazomib, lenalidomide or dexamethasone

### Efficacy

The response to treatment is described in Table 3. The primary endpoint of confirmed VGPR + CR rate was 50.0% (95% CI 31.9–68.1), which was above the threshold rate of 39.0% based on the results of the MM1 study. The ORR was 84.4% (95% CI 67.2–94.7) and the CR rate was 28.1% (95% CI 13.7–46.7), with the stringent CR rate being 25.0% (95% CI 11.5–43.4). In the high-risk and standard-risk cytogenetics subgroups, the ORRs were 72.7% and 90.5%, respectively. In the response-evaluable analysis set, stable disease was demonstrated in 15.6% of patients and no patients had PD as their best response.

**Table 3:** Summary of response to treatment in the response-evaluable analysis set

	Ixazomib + LenDex *N* = 32^a^		
	*n*	%	95% CI
CR	9	(28.1)	(13.7, 46.7)
sCR	8	(25.0)	(11.5, 43.4)
PR	18	(56.3)	(37.7, 73.6)
VGPR	7	(21.9)	(9.3, 40.0)
Overall response (≥ PR)	27	(84.4)	(67.2, 94.7)
VGPR or better (CR + VGPR)	16	(50.0)	(31.9, 68.1)
SD	5	(15.6)	(5.3, 32.8)
PD	0	(0.0)	(0.0, 10.9)

*CI* confidence interval, *CR* complete response, *LenDex* lenalidomide and dexamethasone, *PD* progressive disease, *PR* partial response, *sCR* stringent complete response, *SD* stable disease, *VGPR* very good partial response

^a^ Response-evaluable population: among 34 subjects enrolled in the study, 32 subjects who received at least one dose of study drug, had measurable disease at baseline, and had at least one post-baseline response assessment. Two enrolled patients were not response-evaluable: one subject did not have any evaluable disease at baseline and one was not evaluable for response assessment

The median DOR was not estimable for patients with VGPR or better. At the last assessment, no PD was documented in 78% of patients with CR (*n* = 9), 56% of patients with VGPR or better (*n* = 16) and 48% of patients with PR or better (*n* = 27). The median time to response was 2.9 months (95% CI 1.8–5.1) for VGPR or better and 1.0 months (95% CI 1.0–1.8) for PR or better.

**Fig. 1:**
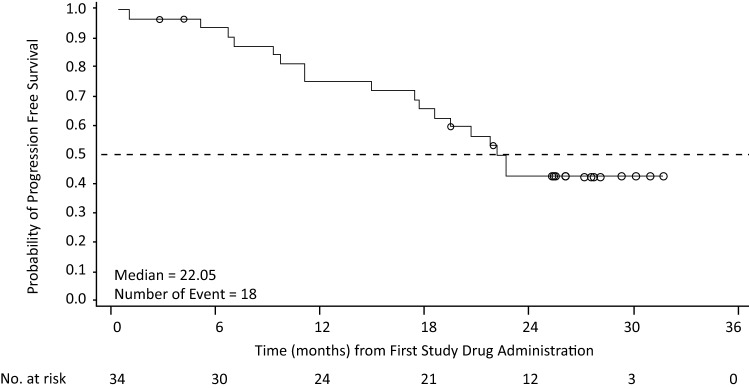
Progression-free survival in the full analysis set

PFS is described in Fig. 1. With a median follow-up of 28.1 months, median PFS was 22.0 months (95% CI 17.3–not evaluable); and 18 patients (53%) had PD, including one death.

At the time of data cutoff, the OS data were not mature, and the median OS was not estimable.

### Safety

The overall summary of treatment-emergent adverse events (TEAEs) is described in Table 4 and the most common TEAEs that were reported in ≥ 10% of patients are described in Table 5. All patients experienced at least one TEAE and drug-related TEAE during the study (*n* = 34 [100%]). Additionally, 85% of patients had at least 1 TEAE of Grade ≥ 3 and 79% of patients had a drug-related TEAE of Grade ≥ 3. The most common TEAEs of Grade ≥ 3 were neutropenia (21%), decreased platelet count (21%), decreased neutrophil count (18%), diarrhea (15%) and maculopapular rash (12%) (Table 6). Two deaths were observed, with one death due to adverse events (AEs) (subarachnoid hemorrhage and subdural hemorrhage) that were associated with a fall and without study drug causality, and another death due to primary disease or its complications after discontinuation of study treatment for PD.

**Table 4:** Overall summary of TEAEs in the safety analysis set

	Ixazomib + LenDex *N* = 34 *n* (%)
Any AE	34 (100)
Grade 3 or higher AE	29 (85)
Drug-related AE	34 (100)
Drug-related Grade 3 or higher AE	27 (79)
SAE	12 (35)
Drug-related SAE	10 (29)
AEs resulting in any study drug dose reduction	26 (76)
AEs resulting in any study drug dose modification^a^	29 (85)
AEs resulting in any study drug discontinuation^b^	11 (32)
On-study deaths^c^	1 (3)

Dose reduction, dose modification and study drug discontinuation may have been in relation to any of the three drug regimens. On-study deaths were defined as deaths that occurred within 30 days of the last dose of study drug

*AE* adverse event, *LenDex* lenalidomide and dexamethasone, *SAE* serious adverse event, *TEAE* treatment-emergent adverse event

^a^Dose modification included dose reduction, dose increase, dose delay and dose discontinuation

^b^Study drug discontinuation may have been in relation to ≥ 1 of the three study drugs

^c^The subject died of subarachnoid hemorrhage and subdural hemorrhage during the study. The event was assessed to be associated with a fall; causality with the study drug was ruled out

**Table 5:** The most common (≥ 10%) TEAEs in the safety analysis set

	Ixazomib + LenDex *N* = 34 *n* (%)
Patients with at least 1 AE	34 (100)
Constipation	17 (50)
Upper respiratory tract infection	16 (47)
Diarrhea	14 (41)
Rash	12 (35)
Nasopharyngitis	11 (32)
Nausea	10 (29)
Platelet count decreased	9 (26)
Influenza	8 (24)
Neutropenia	8 (24)
Chilblains	7 (21)
Dysgeusia	7 (21)
Fall	7 (21)
Peripheral sensory neuropathy	7 (21)
Bronchitis	6 (18)
Neutrophil count decreased	6 (18)
Dental caries	5 (15)
Malaise	5 (15)
Edema peripheral	5 (15)
Pharyngitis	5 (15)
Maculopapular rash	5 (15)
Vomiting	5 (15)
Back pain	4 (12)
Dry skin	4 (12)
Hyperglycemia	4 (12)
Oropharyngeal pain	4 (12)
Pain in extremity	4 (12)
Pneumonia	4 (12)
Pyrexia	4 (12)
Stomatitis	4 (12)
Thrombocytopenia	4 (12)
Urticaria	4 (12)

*AE* adverse event, *LenDex* lenalidomide and dexamethasone, *TEAE* treatment-emergent adverse event

**Table 6:** Grade 3 or higher TEAEs in the safety analysis set

	Ixazomib + LenDex *N* = 34 *n* (%)
Neutropenia	7 (21)
Platelet count decreased	7 (21)
Neutrophil count decreased	6 (18)
Diarrhea	5 (15)
Maculopapular rash	4 (12)
Erythema multiforme	3 (9)
Pneumonia	3 (9)
White blood cell count decreased	3 (9)
Alanine aminotransferase increased	1 (3)
Anemia	1 (3)
Aspartate aminotransferase increased	1 (3)
Blood creatine phosphokinase increased	1 (3)
Cellulitis	1 (3)
Fall	1 (3)
Femoral neck fracture	1 (3)
Hyperglycemia	1 (3)
Hypertension	1 (3)
Hypophosphatemia	1 (3)
Hypotension	1 (3)
Leukemia	1 (3)
Lipase increased	1 (3)
Loss of consciousness	1 (3)
Lung neoplasm malignant	1 (3)
Lymphocyte count decreased	1 (3)
Malignant neoplasm of unknown primary seta^a^	1 (3)
Edema peripheral	1 (3)
Osteonecrosis of jaw	1 (3)
Pharyngitis	1 (3)
Pneumonitis	1 (3)
Rash generalized	1 (3)
Macular rash	1 (3)
Sebaceous nevus	1 (3)
Subarachnoid hemorrhage	1 (3)
Subdural hemorrhage	1 (3)
Thrombocytopenia	1 (3)
Tooth infection	1 (3)
Vomiting	1 (3)

Toxicity grade defined according to the National Cancer Institute Common Terminology Criteria for Adverse Events version 4.03

*LenDex* lenalidomide and dexamethasone, *TEAE* treatment-emergent adverse event

^a^Reported during the follow-up period

Serious adverse events (SAEs) were experienced by 35% of patients (*n* = 12), with 29% experiencing drug-related SAEs. The most common SAEs were pneumonia (9%), diarrhea (6%) and fall (6%), while the most common drug-related SAEs were pneumonia (9%) and diarrhea (6%).

Overall, 32% of patients had at least one TEAE leading to the discontinuation of one or more of the three study drugs; the most common AE was neutropenia, which was reported in two patients. Additionally, 76% of patients had at least one TEAE resulting in dose reduction of any study drug, with the most common AE being maculopapular rash, which was reported in four patients. Two patients were diagnosed with a second malignancy while on study treatment: one patient was reported as having acute myeloid leukemia and the other had malignant lung neoplasm. Both of these AEs were serious, considered to be related to study treatment and resulted in discontinuation of study treatment. Patients were specifically followed up for second malignancies because of the increased risk with lenalidomide [20, 21].

TEAEs occurring in ≥ 20% of patients were constipation (50%), upper respiratory tract infection (47%), diarrhea (41%), rash (35%), nasopharyngitis (32%), nausea (29%), platelet count decreased (26%), influenza (24%), neutropenia (24%), chilblains (21%), dysgeusia (21%), fall (21%) and peripheral sensory neuropathy (21%).

Drug-related TEAEs occurring in ≥ 20% of patients were diarrhea (38%), constipation (38%), rash (29%), platelet count decreased (26%), nausea (24%), peripheral sensory neuropathy (21%), dysgeusia (21%) and neutropenia (21%). Drug-related TEAEs of Grade ≥ 3 occurring in ≥ 10% of patients were platelet count decreased (21%), neutrophil count decreased (18%), neutropenia (18%), diarrhea (15%) and maculopapular rash (12%).

A summary of TEAEs of clinical importance is presented in Table 7. The TEAEs of clinical importance were neutropenia, thrombocytopenia, heart failure, arrhythmia, myocardial infarction, nausea, vomiting, diarrhea, rash, maculopapular rash, urticaria, erythema multiforme, generalized rash, macular rash, peripheral sensory neuropathy and peripheral neuropathy.

**Table 7:** Summary of TEAEs of clinical importance^a^ in the safety analysis set

*N* = 34, *n* (%)	Any Grade		≥ Grade 3		Treatment/response			
	Adverse event	Drug related	Adverse event	Drug related	Dose discontinuation	Dose reduction	Patient withdrawn from study^b^	Next cycle delayed
*Blood toxicity*								
Neutropenia	14 (41)	13 (38)	13 (38)	12 (35)	3 (9)	6 (18)	1 (3)	10 (29)
Thrombocytopenia	13 (38)	13 (38)	8 (24)	8 (24)	3 (9)	4 (12)	2 (6)	5 (15)
*Cardiac disorders*								
Cardiac failure	1 (3)	0	0	0	0	0	0	0
*Gastrointestinal disorders*								
Nausea	10 (29)	9 (26)	0	0	0	0	0	0
Vomiting	5 (15)	5 (15)	1 (3)	1 (3)	0	1 (3)	1 (3)	0
Diarrhea	14 (41)	13 (38)	5 (15)	5 (15)	0	2 (6)	4 (12)	0
*Dermatologic disorders*	22 (65)	20 (59)	9 (26)	8 (24)	0	8 (24)	11 (32)	2 (6)
Rash	12 (35)	10 (29)	0	0	0	0	2 (6)	0
Maculopapular rash	5 (15)	5 (15)	4 (12)	4 (12)	0	4 (12)	4 (12)	0
Urticaria	4 (12)	3 (9)	0	0	0	0	0	0
Erythema multiforme	3 (9)	3 (9)	3 (9)	3 (9)	0	2 (6)	3 (9)	0
Generalized rash	1 (3)	1 (3)	1 (3)	1 (3)	0	1 (3)	1 (3)	1 (3)
Macular rash	1 (3)	1 (3)	1 (3)	1 (3)	0	1 (3)	1 (3)	1 (3)
*Peripheral neuropathy*								
Peripheral sensory neuropathy	7 (21)	7 (21)	0	0	0	1 (3)	0	1 (3)
Peripheral neuropathy	1 (3)	1 (3)	0	0	0	0	0	0

*TEAE* treatment-emergent adverse event

^a^TEAEs of clinical importance was not based on a safety signal in the review of the clinical data; rather, they were considered of clinical importance owing to other factors that included, but were not limited to: (1) identification by searches of the clinical database considering the context of the intended patient population; (2) common adverse reactions for lenalidomide; (3) AEs reported at higher rates both across ixazomib clinical trials and within the MM1 study; and (4) adverse reactions reported with the commercially available PIs bortezomib and carfilzomib

^b^Some patients withdrew from the study due to multiple TEAEs

AEs of clinical importance reported in this study were similar to those reported in the global MM1 study. No previously unknown safety concerns were identified in this study.

## Discussion

In this study, all demographics and other baseline characteristics except race were similar to those of the MM1 study [17]. The efficacy of ixazomib when administered with LenDex in Japanese RRMM patients was similar to that in the intent-to-treat (ITT) population of the MM1 study. The confirmed VGPR + CR rate was 50.0% and the point estimate demonstrated in this study exceeded the threshold rate of 39.0%, which was based on the results of the MM1 study. The confirmed ORR and CR rates in this study (84.4% and 28.1%, respectively) were numerically better than those in the MM1 study (78% and 12%, respectively). The ORR for the high-risk subgroup was 72.7% and 90.5% for the standard-risk subgroup, although the number of patients with high-risk cytogenetics was small in this study. The favorable result for the primary efficacy endpoint was supported by the findings from the secondary efficacy endpoints, including PFS, DOR and TTP.

The safety profile of ixazomib when administered with LenDex in Japanese RRMM patients was similar to that in the ITT population of the MM1 study [17]. No new safety concerns were identified in this study, and the overall safety profile of ixazomib plus LenDex showed that this combination was well tolerated.

Diarrhea was the most commonly reported gastrointestinal AE of clinical importance. While antidiarrheal agents are not recommended for prophylactic use, they should be used appropriately as symptomatic treatment. In this study, antidiarrheal agents were administered if infectious causes were excluded.

Dermatological disorders were reported in 65% of patients, while they did not result in any patient discontinuing the study treatment, 24% of patients had their doses of ixazomib and/or lenalidomide reduced, and 32% had their doses of ixazomib and/or lenalidomide withheld. This corresponds with the incidence of rash observed in the Japanese subgroup enrolled in the MM1 study (70% reported rash) and higher than that reported in the overall population in the MM1 study (51% reported rash) [17], suggesting a slightly higher frequency of skin disorders developing in the Japanese population. However, this AE was manageable either by dose reduction or supportive care.

This study employed a dose modification guideline for rash that was modified from the MM1 study [17], and consistent with the Japanese package insert (i.e., ixazomib and lenalidomide were withheld in the event of Grade 2 rash not manageable by supportive care, until it recovered to Grade 1 or better). The successful management of rash may have contributed to the high relative dose intensity of lenalidomide while also maximizing exposure to ixazomib.

Supportive care and dose modification that are dependent on the patient’s AEs seem an effective approach for continuous treatment, resulting in better outcomes. This is supported by the results in this study, with patients receiving a median of 20 treatment cycles and showing good responses.

This study has several clinical limitations. Its single-arm, open-label design may bias the interpretation of the study results. Secondly, the sample size and inclusion/exclusion criteria of the study limit the generalizability of its results. For example, 82% of patients were international staging system (ISS) stage I, 74% had an ECOG performance status of 0, 62% only had one prior line of therapy, 91% had relapsed disease and 68% had undergone ASCT. Hence, the generalizability of these results to patients with more advanced stage, poorer performance status or multiple lines of therapy, those with refractory disease or ASCT-ineligible patients may be limited.

In conclusion, ixazomib, when administered with LenDex, demonstrated efficacy in achieving a 50% rate of confirmed VGPR or better in Japanese patients with RRMM, which was higher than the 39.0% threshold rate. The results demonstrated in this study are comparable with those in the MM1 study [17]. Likewise, the overall safety profile of ixazomib when administered with LenDex showed that this combination was well tolerated in this population.

## Supplementary Information

Below is the link to the electronic supplementary material.

Supplementary file1 (DOCX 54 KB)

## Data Availability

The authors do not plan to share individual participant’s data supporting the results reported in this article because informed consent about external data sharing has not been obtained in any patient in the study due to investigator sites’ policy. The redacted study protocol, redacted statistical analysis plan, in this article will be made available, within 3 months from initial request, to researchers who provide a methodologically sound proposal.
