# Supplementary material for: A phase 2, open-label, multicenter study of ixazomib plus lenalidomide and dexamethasone in adult Japanese patients with relapsed and/or refractory multiple myeloma
Source: Int J Clin Oncol. 2021 Oct 2;27(1):224–33. doi: 10.1007/s10147-021-02030-7 (PMC8732884; doi:10.1007/s10147-021-02030-7)
Supplement: Supplementary file 1 — Supplementary file1 (DOCX 54 KB) [file 10147_2021_2030_MOESM1_ESM.docx]

**Article title: A Phase 2, Open-Label, Multicenter Study of Ixazomib Plus Lenalidomide and Dexamethasone in Adult Japanese Patients with Relapsed and/or Refractory Multiple Myeloma**

**Journal name: International Journal of Clinical Oncology**

**Authors:** Shinsuke Iida, Tohru Izumi, Takuya Komeno, Yasuhito Terui, Takaaki Chou, Takashi Ikeda, Deborah Berg, Shinichi Fukunaga, Kenkichi Sugiura, Makoto Sasaki

**Corresponding author**: Shinsuke Iida

Affiliation: Department of Hematology and Oncology, Nagoya City University Graduate School of Medical Sciences, Nagoya, Aichi, Japan

Email: [iida@med.nagoya-cu.ac.jp](mailto:iida@med.nagoya-cu.ac.jp)

**Online Resource 1.** Additional inclusion criteria and exclusion criteria for study population.

| **Additional inclusion criteria** |
| --- |
| - Patients who received a prior allogenic transplant must have no active graft-versus-host disease. - Absolute neutrophil count ≥1,000/mm^3^, hemoglobin ≥8 g/dl, platelet count ≥75,000/mm^3^, total bilirubin ≤1.5 × the upper limit of the normal range (ULN), alanine aminotransferase and aspartate aminotransferase ≤3 × ULN, and calculated creatinine clearance ≥30 ml/min. - Female patients had to be postmenopausal for 24 months prior to screening, or were surgically sterile, or have a negative pregnancy test within 10–14 days and within 24 hours prior to first cycle of lenalidomide, and agree to practice birth control, with ongoing pregnancy testing from 28 days before treatment through to 90 days after the last study dose. - Male patients, even if surgically sterile, had to avoid sexual intercourse for 90 days after the last study dose, or practice effective barrier contraception during treatment and 90 days after the last study dose. |
| **Additional exclusion criteria** |
| - Not fully recovered (i.e., Grade ≤1 toxicity) from the effects of prior chemotherapy (except for hair loss) regardless of the interval since the last treatment. - Major surgery or radiotherapy within 14 days before enrollment. - Central nervous system involvement. - Infection requiring systemic antibiotic therapy or other serious infection within 14 days before enrollment. - Rash or pruritus requiring systemic medication within 14 days before enrollment. - Diagnosis of Waldenstrom’s macroglobulinemia; polyneuropathy, organomegaly, endocrinopathy, monoclonal gammopathy and skin changes (POEMS) syndrome; plasma cell leukemia; primary amyloidosis; myelodysplastic syndrome; or myeloproliferative syndrome. - Evidence of current uncontrolled cardiovascular conditions, including uncontrolled hypertension, uncontrolled cardiac arrhythmias, symptomatic congestive heart failure, unstable angina or myocardial infarction within the past 6 months before enrollment. - Systemic treatment with strong cytochrome P450, family 3, subfamily A (CYP3A) inducers (rifampicin, carbamazepine, phenytoin), or St John’s wort within 14 days before enrollment. - Ongoing or active systemic infection, known HIV positive, known hepatitis B surface antigen seropositive or known hepatitis C virus RNA positive. - Comorbid systemic illnesses or other severe concurrent disease, which would have interfered significantly with the proper assessment of safety and toxicity of the prescribed regimens. - Psychiatric illness/social situation that would have limited compliance with study requirements. - Known allergy to any of the study medications, their analogs, or excipients in the various formulations of any agent. - Inability to swallow oral medication, inability or unwillingness to comply with the drug administration requirements, or a gastrointestinal condition that could interfere with the oral absorption or tolerance of treatment. - Diagnosed or treated for another malignancy within 2 years before enrollment or previously diagnosed with another malignancy and had any evidence of residual disease. Patients with non-melanoma skin cancer or carcinoma in situ of any type were not excluded if they had undergone complete resection. - Patients who had participated in the clinical trial of ixazomib or had been treated with ixazomib. |

**Online Resource 2.** Definition of secondary endpoints.

Overall survival (OS): the time from the date of the first study drug administration to the date of death

Overall response rate (ORR): the rate of PR or better

Duration of response (DOR): the time from the date of the first documentation of response to the date of the first documentation of PD

Time to progression (TTP): the time from the date of the first study drug administration to the date of the first documentation of PD

Safety includes treatment-emergent adverse events (TEAEs) of clinical importance, laboratory parameters and vital signs.
